# Liposomes Containing Amaranth Unsaponifiable Matter and Soybean Lunasin Suppress ROS Production in Fibroblasts and Reduced Interleukin Production in Macrophages

**DOI:** 10.3390/ijerph191811678

**Published:** 2022-09-16

**Authors:** Gloria Dávila-Ortiz, Erick Damian Castañeda-Reyes, Carlos Ignacio Juárez-Palomo, María de Jesús Perea-Flores, Ricardo Pérez-Pastén-Borja, Yazmín Karina Márquez-Flores, Elvira González de Mejía

**Affiliations:** 1 Unidad Profesional Adolfo López Mateos, Departamento de Ingeniería Bioquímica, Escuela Nacional de Ciencias Biológicas, Instituto Politécnico Nacional, Campus Zacatenco, Madero, Ciudad de México 07738, Mexico; 2 Department of Food Science and Human Nutrition, University of Illinois, Urbana-Champaign, IL 61801, USA; 3 Unidad Profesional Adolfo López Mateos, Centro de Nanociencias y Micro y Nanotecnologías, Instituto Politécnico Nacional (IPN), Av. Luis Enrique Erro s/n, Zacatenco, Alcaldía Gustavo A. Madero, Ciudad de México 07738, Mexico; 4 Unidad Profesional Adolfo López Mateos, Departamento de Farmacia, Escuela Nacional de Ciencias Biológicas, Instituto Politécnico Nacional, Campus Zacatenco, Madero, Ciudad de México 07738, Mexico

**Keywords:** liposomes, interleukin-6, tumor necrosis factor-α, ROS-production, amaranth squalene, soybean lunasin, inflammation

## Abstract

Inflammation is a normal response in defense to agents that may cause damage to the human body. When inflammation becomes chronic, reactive oxygen species (ROS) are produced; which could lead to diseases such as cancer. The aim was to assess liposomes’ antioxidant and anti-inflammatory capacity loaded with amaranth unsaponifiable matter and soybean lunasin (UM + LunLip) in an in vitro model using fibroblasts and macrophages. To evaluate ROS production, fibroblasts CHON-002 ABAP were added to promote ROS production; and the cells were treated with UM + LunLip. For inflammation markers production, lipopolysaccharides (LPS)-stimulated RAW 264.7 and peritoneal macrophages were treated with empty liposomes (EmLip), liposomes loaded with unsaponifiable matter (UMLip), liposomes loaded with lunasin (LunLip), and UM + LunLip. ROS production was significantly decreased by 77% (*p* < 0.05) when fibroblasts were treated with UM + LunLip at 2 mg lunasin/mL compared with the control treated with ABAP. Treatment with UMLip was the most effective in reducing tumor necrosis factor-α (71–90%) and interleukin-6 (43–55%, *p* < 0.001). Both liposomes containing unsaponifiable matter (UMLip and UM + LunLip) were more effective than EmLip or LunLip. In conclusion, amaranth unsaponifiable matter-loaded liposomes are effective in decreasing pro-inflammatory cytokine production.

## 1. Introduction

Inflammation is a defense response against damaged tissue and foreign bodies [1]; it involves the activation, recruitment, and action of cells of the innate and adaptative immunity [2]. There are two types of inflammation: acute and chronic. Acute inflammation aims to eliminate agents that may cause damage, such as microbes or dead cells; once eliminated, the inflammation decreases [1]. Chronic inflammation predisposes cancer development and promotes tumorigenesis [1,2].

Under normal health conditions, inflammation is an essential process for maintaining homeostasis [2]. Therefore, its regulation is required; once the emergency has been addressed, the reaction ceases, and health restoration can proceed (acute inflammation). However, there are persistent transmissible pathological situations, such as microbial infections or non-transmissible disorders including diabetes, arthritis, and obesity, among others, which keep the organism in a state of sustained inflammation (chronic inflammation) [1]. Unattended chronic inflammation predisposes tumor progression and promotes cancer over time [1,2].

The inflammatory process can be produced due to the overexpression of certain cytokines, such as interleukin (IL)-1, IL-6, and tumor necrosis factor (TNF)-α, among other factors; including the interaction through membrane-associated molecules of B and T lymphocytes, myeloid, epithelial, endothelial, and muscle cells, as well as fibroblasts and adipocytes [3].

IL-6 is produced by monocytes and macrophages when infection or tissue injury occurs [4,5]. Following IL-6 production, it enters the bloodstream and induces acute phase proteins, such as the C-reactive protein, serum amyloid A, and others [6]. Thus, the presence of IL-6 induces fever and increases C-reactive protein concentration in the human body [7]. When the host is relieved of stress, the production of IL-6 ceases [5].

IL-6 is an important marker expressed during chronic inflammatory disease [3] since chronic inflammation is exacerbated by the dysregulation of IL-6 production [6]. Chronic inflammation stimulates the production of reactive nitrogen/oxygen species (RNS/ROS) that can produce DNA damage in organs during the inflammatory process, leading to aggressive cancer development [8].

Some lipidic compounds can ameliorate inflammation; for instance, polyunsaturated fatty acids, such as oleic acid, act as a precursor of lipids mediators that regulate inflammation and immune response [9].

Amaranth oil contains a high concentration of unsaturated lipids; linoleic acid is the most abundant fatty acid, followed by oleic acid; amaranth unsaponifiable matter includes tocopherols, tocotrienols, sterols, and squalene (SQ), among others [10,11]. There are not too many reports about SQ and its anti-inflammatory activity. Nevertheless, the anti-inflammatory effects of SQ have been reported in adipose tissue [12], monocytes [13], and proinflammatory M1 macrophages [14].

On the other hand, it has been reported that lunasin, a soybean peptide, possibly diminishes the obesity-induced inflammatory response. Lunasin effectively decreased inflammation markers’ expression in LPS-stimulated macrophages RAW 264.7 and 3T3-L1 adipocytes, as well as the co-culture of both cell lines [15]. It has been suggested that the anti-inflammatory effect of lunasin is due to the suppression of the nuclear factor kappa-light-chain-enhancer of activated beta cells (NF-κβ) [16].

We hypothesized that liposomes prepared with dioleoyl phosphatidylcholine (DOPC) and dioleoyl phosphatidylglycerol (DOPG), and loaded with amaranth unsaponifiable matter as squalene source and soybean lunasin (UM + LipLun) can decrease the expression of IL-6 and TNF-α in RAW 264.7 and mouse peritoneal macrophages after stimulation with LPS; as well as the expression of ROS in CHON-002 fibroblasts.

The objective was to evaluate the production of IL-6 and TNF-α after the stimulation with LPS of RAW 264.7 and peritoneal macrophages; as well as the ROS production after stimulation with 2,2′-Azobis (2-methylpropionamidine) dihydrochloride (ABAP) using UM + LunLip as treatments.

## 2. Materials and Methods

### 2.1. Material

Cell line CHON-002 (ATCC CRL-2847) was purchased from the American Type Culture Collection (Manassas, VA, USA). Cell line RAW 264.7 was kindly donated by Dr. Diego Luna Vital. Dulbecco’s modified eagle medium (DMEM, 12800-017) was purchased from Gibco Life Technologies (Grand Island, NY, USA). Furthermore, 5-(and 6-) chloromethyl-2′,7′-dichlorodihydrofluorescein diacetate, acetyl ester (CM-H_2_DCFDA, C6827) was acquired from Invitrogen (Carlsbad, CA, USA). Mouse tumor necrosis factor-α (TNF-α), ELISA kit (RAB0477-1KT), and mouse interleukin (IL)-6 kit (RAB0308-1KT) were purchased from Sigma-Aldrich (St. Louis, MO, USA). All the other reagents were purchased from Sigma-Aldrich unless otherwise noted.

### 2.2. Methods

#### 2.2.1. Liposomes Preparation

Liposomes were prepared as previously described by Castañeda-Reyes et al. [11]. Characterization regarding encapsulation efficiency, zeta potential, and morphology was previously analyzed as well.

#### 2.2.2. Cell Culture Procedures

Both cell lines (fibroblasts CHON-002 and mouse macrophages RAW 264.7) were grown in DMEM with sodium bicarbonate (1.5 g/mL) supplemented with 10% or 5% fetal bovine serum (FBS). Peritoneal macrophages were kept in Roswell Park Memorial Institute Medium (RPMI), 1640 phenol red-free medium supplemented with 10% or 5% FBS. All the cells were maintained or incubated at 37 °C using 95% air/5% CO_2_.

#### 2.2.3. Thiazolyl Blue Tetrazolium Bromide (MTT) Cell Viability Assay

Aiming to investigate liposome cytotoxicity after 24 h exposure, an MTT cell viability assay was performed. All the cells (CHON-002, RAW 264.7, and the peritoneal macrophages; isolation is described below) were seeded in 96-well plates with 100 μL of 4 × 10^5^ cells/mL for CHON-002 and 100 μL of 1 × 10^6^ cells/mL for both peritoneal macrophages and RAW 264.7. MTT was used with a concentration of 0.5 mg/mL, as described by Liang et al. [17].

#### 2.2.4. Cellular Antioxidant Activity (CAA)

Cellular antioxidant activity (CAA) was measured according to Sinisgalli et al. [18], with some modifications. An aliquot of 100 μL of CHON-002 (4x10^5^ cells/mL) was seeded in a 96-well plate and incubated overnight at the conditions specified before. Once attached, the cells were washed twice with Hank’s saline buffered solution (HSBS) and treated with 50 μL of different concentrations of liposomes loaded with soybean lunasin and amaranth unsaponifiable matter (0.5–2 mg lunasin/mL); or quercetin (0–0.5 mM) and 50 μL of 0.02 μM CM-H_2_DCFDA per 1 h at 37 °C and 95% air/5% CO_2_. Following three washes with HBSS, the cells were stimulated with 600 μM 2,2′-Azobis(2-methylpropionamidine) dihydrochloride (ABAP); and fluorescence kinetics were immediately recorded every 5 min using a Varioskan lux (Thermo Fisher Scientific, Inc., Waltham, MA, USA).

#### 2.2.5. Cytokines Expression in Mouse Peritoneal Macrophages

The laboratory animal procedures were conducted according to the Mexican Official Guidelines NOM-062-ZOO-1999, “Technical specifications for the production, care, and use of laboratory animals”. The Research Ethics Committee of the National School of Biological Sciences, I.P.N., reviewed and approved the research protocol (number ZOO-007-2022).

Four C57BL6/J mice were purchased from the college of sciences’ biotherium, U.N.A.M. For peritoneal extraction, we followed the methodology proposed by Muneoka et al. [19] administering intraperitoneally 1 mL of 10% thioglycolate; after euthanasia, the peritoneum was washed with ice-cold PBS. The suspension was centrifuged twice per 5 min at 1200 RPM; the PBS was changed after the first centrifugation and was replaced for RPMI 1640 supplemented with 10% FBS after the second centrifugation; 1 mL of the isolated macrophages were seeded in 24-well plates at 1 × 10^6^ and incubated 2 h at 37 °C in 95% air/5% CO_2_; 1 mL of treatments with liposomes were loaded with amaranth unsaponifiable matter and lunasin (UM + LunLip) at a concentration of 2 mg lunasin/mL; liposomes were loaded with lunasin (LunLip) at a concentration of 2 mg lunasin/mL; and both amaranth unsaponifiable matter liposomes (UMLip) and empty liposomes (EmLip) were applied in a concentration equivalent to the UM + LunLip to make them comparable. The control group was grown in a fresh medium without liposomes or LPS, whereas the control LPS group was only stimulated with LPS to create a reference group with the highest cytokine production. The treatments were diluted in RPMI 1640 supplemented with 5% FBS. After 30 min incubation, the cells were stimulated with 1 μg/mL lipopolysaccharide (LPS) from *Escherichia coli* for 18 h. It was reported that after 16–18 h of LPS stimulation, cytokines can be detected [20]. The culture supernatant was collected and stored at −80 °C for further analysis.

#### 2.2.6. Cytokines Expression in RAW 264.7

The procedure for LPS stimulation was performed as described in the previous section using the same conditions and treatments.

#### 2.2.7. Cytokines Interleukin (IL)-6 and Tumor Necrosis Factor (TNF)-α

The protocols provided by the kits developer were strictly followed using the samples collected after LPS stimulation of peritoneal macrophages and RAW 264.7 mouse macrophages.

#### 2.2.8. Statistical Analysis

GraphPad Prism 8 (GraphPad Software 9, Inc., Sand Diego, CA, USA, 2021) was used. Experimental data were analyzed by one-way analysis of variance with the Dunnett post hoc test. A *p* < 0.05 was considered significant. All the data were expressed as mean ± standard deviation.

## 3. Results and Discussion

**Figure 1:**
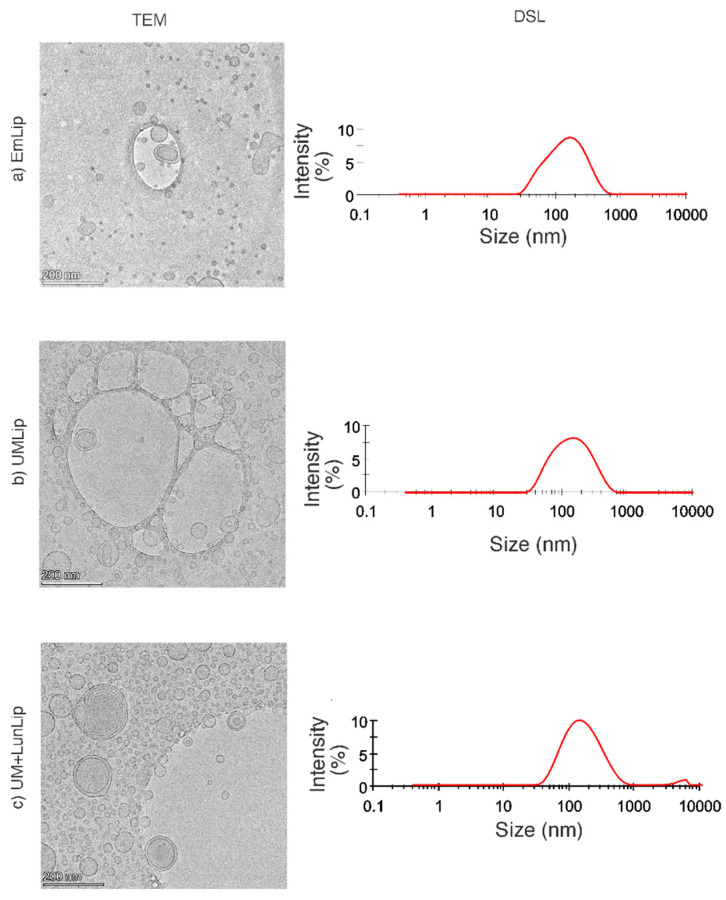
Transmission electron microscopy (TEM) micrographs and particle size distribution from dynamic light scattering (DSL): (**a**) empty liposomes (EmLip); (**b**) liposomes loaded with amaranth unsaponifiable matter (UMLip, 1.10 mg/mL) containing DOPC-DOPG 15.27 mg/mL; and (**c**) liposomes loaded with unsaponifiable matter (1.10 mg/mL) and soybean lunasin (4 mg/mL). The bilayer of all the liposomes contained dioleoyl phosphatidylcholine (DOPC)/dioleoyl phosphatidyl glycerol (DOPG) 15.27 mg/mL. Bars = 200 nm. The larger structures are part of the holey carbon film, which contains unobstructed regions to reduce noise from the background. A picture of the holey carbon coat can be found at the supplier’s website: https://www.2spi.com/category/grids-custom-holey-carbon/, accessed 17 August 2022.

The liposomes that were used in the present research are shown in Figure 1; the liposome size was from 121.30 to 128.60 nm, with an encapsulation efficiency of 59–82% and a zeta potential from −75.91 to −79.21 mV [11]. According to the zeta potential, the liposome solutions were stable. The range to consider a particle solution as stable is greater than 35 mV or lower than −35 mV; when the zeta potential is closer to 0, the particles will aggregate [21].

### 3.1. Liposomes Decreased the Reactive Oxygen Species (ROS) Expression in CHON-002 Fibroblasts When Cellular Antioxidant Capacity Was Assessed

Aiming to establish the concentration of lunasin to be used in the cytokine expression experiments, we evaluated the ROS expression in fibroblasts CHON-002 using CM-H_2_DCFDA and ABAP.

Once the non-fluorescent probe CM-H_2_DCFDA diffuses into the cells, the thiols may interact with chloromethyl groups. After the interaction, the acetate groups will be hydrolyzed by esterases to dichlorofluorescein (DCFH); which will enhance the intracellular retention. The non-fluorescent DCFH will be oxidized by ROS into a highly fluorescent dichlorofluorescein (DCF) [22].

**Figure 2:**
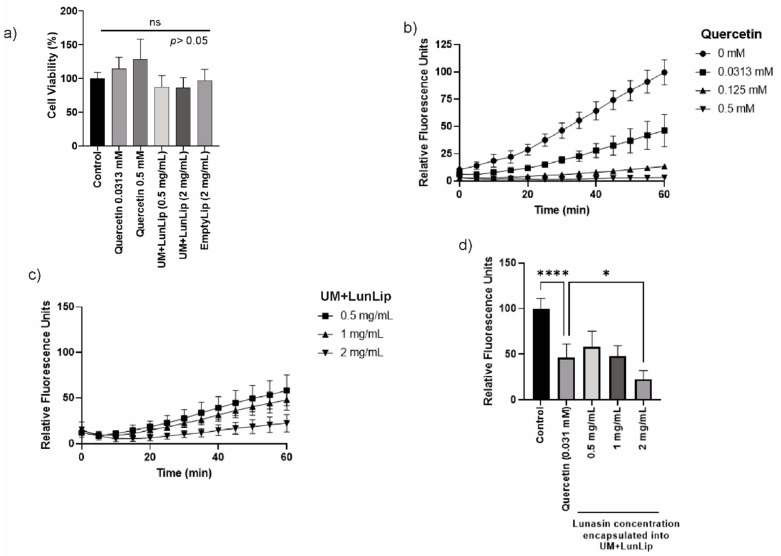
Liposomes prepared with dioleoyl phosphatidylcholine and dioleoyl phosphatidylglycerol, and loaded with soybean lunasin and amaranth unsaponifiable matter (UM + LipLun) decreased ROS production when ABAP was applied to the CHON-002 fibroblasts. (**a**) Cell viability of CHON-002 after treatment with empty liposomes (EmLip), UM + LunLip, and quercetin. (**b**) Relative fluorescence of dichlorofluorescein (DCF) after treatment with different concentrations of quercetin (0–0.5 mM). (**c**) Relative fluorescence of DCF after treatment with different concentrations of UM + LunLip (0.5–2 mg lunasin/mL). (**d**) ROS production after 1 h treatments with different concentrations of UM + LunLip; 0.031 mM quercetin was used as the antioxidant control and non-treated cells as the control. The ROS production in cells (from a to c) was stimulated with 600 μM ABAP. The data are present as the mean of two independent replicates; each was measured in triplicate. Data are means ± standard deviation. * *p* < 0.05; **** *p* < 0.0001. ns, not statistically different.

Since cell viability was not affected after treatment with neither the higher dosage of UM + LunLip nor quercetin or EmLip (Figure 2a), the reduction in ROS production will be due to the treatments and not because the cell confluency was affected regarding any cytotoxic effect. The kinetics (Figure 2b,c) showed that the higher concentration of both quercetin and UM + LunLip decreased the ROS production to lower than the dosage of the treatments. After 1 h (Figure 2d), the ROS production was decreased by 53% in 0.03 mM quercetin compared to the untreated control; and by 77% when UM + LunLip was applied at 2 mg lunasin/mL after ABAP application compared with the untreated control. Due to these results, a concentration of 2 mg/mL UM + LunLip was selected for the cytokine expression experiments.

ABAP is an oxidant that damages the cells by peroxyl radical production. These radicals affect the cell membrane [23], initiating lipid peroxidation in unsaturated fatty acids [24]. Lipid peroxidation affects the integrity, fluidity, and function of biological membranes. Lipid peroxidation is a self-propagating chain reaction; the initial oxidation of a few lipids could lead to tissue damage [25].

Due to the compounds naturally found in amaranth unsaponifiable matter, such as squalene, tocopherols, sterols, stigmasterol, among others [26], and the liposome bilayer (unsaturated fatty acids) as well as the decrement observed on ROS production (Figure 2d), the lipid peroxidation chain reaction induced by the addition of ABAP in cells could be diminished by the UM + LunLip. It was shown that 50 μM of both squalene and α-tocopherol decreased the production of ROS after 24 h of MCF10A breast epithelial cells exposure [27].

The production of ROS resulting from oxidative stress is related to inflammatory diseases [28]. It was reported that lipid nanoparticles prepared using squalene and adenosine conjugation, and loaded with α-tocopherol showed that nanoparticles decreased both inflammation and ROS generation [29].

### 3.2. UM + LunLip Inhibited the Production of Pro-Inflammatory Markers in RAW 264.7 and Peritoneal Macrophages

Inflammation is an important factor in cancer since the inflammatory response can lead to DNA damage, leading to tumor initiation/progression [30]. Inflammation can be stimulated by lipopolysaccharides. LPS interacts with macrophages since they are Toll-like receptor 4 (TLR4)-expressing host cells. The TLR4 activation produces a dysregulation of pro-inflammatory cytokines, including IL-6 and TNF-α, and ROS production [19,31].

**Figure 3:**
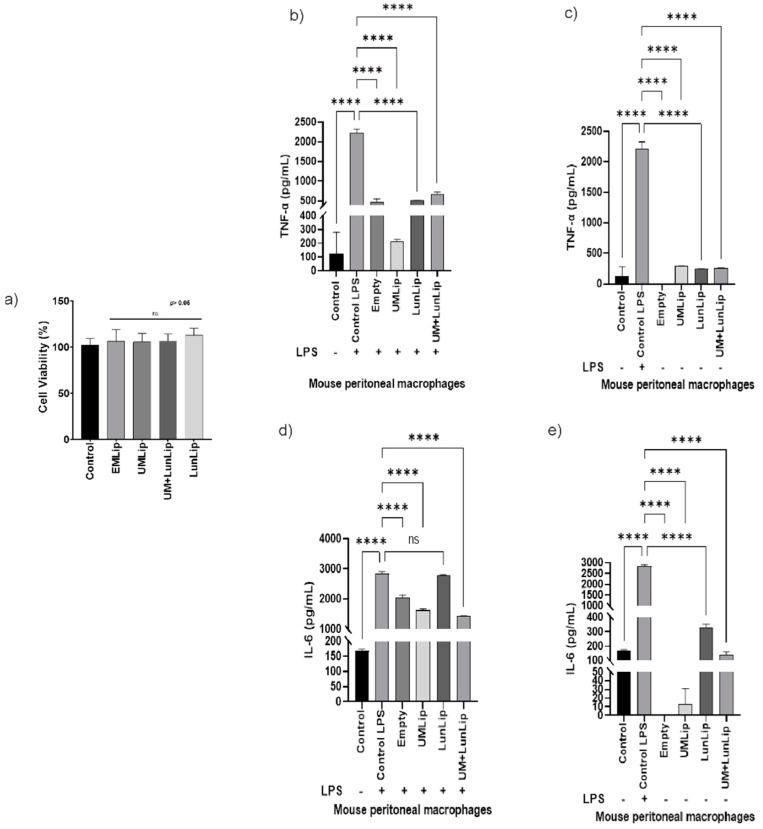
Liposomes prepared with dioleoyl phosphatidylcholine and dioleoyl phosphatidylglycerol, and loaded with soybean lunasin and amaranth unsaponifiable matter (UM + LipLun) decreased TNF-α and IL-6 production after LPS stimulation of peritoneal macrophages. (**a**) Cell viability of peritoneal macrophages after treatment with empty liposomes (EmLip), unsaponifiable matter-loaded liposomes (UMLip), lunasin-loaded liposomes (LunLip), and UM + LunLip. (**b**) TNF-α expression after treatment with liposomes to LPS-stimulated peritoneal macrophages. (**c**) TNF-α expression after treatment with liposomes to non-LPS-stimulated peritoneal macrophages. (**d**) IL-6 expression after treatment with liposomes to LPS-stimulated peritoneal macrophages. (**e**) IL-6 expression after treatment with liposomes to non-LPS-stimulated peritoneal macrophages. All the treatments consisted of 2 mg lunasin/mL or its equivalent in UMLip and EmLip **** *p* < 0.0001. ns, not statistically different.

**Figure 4:**
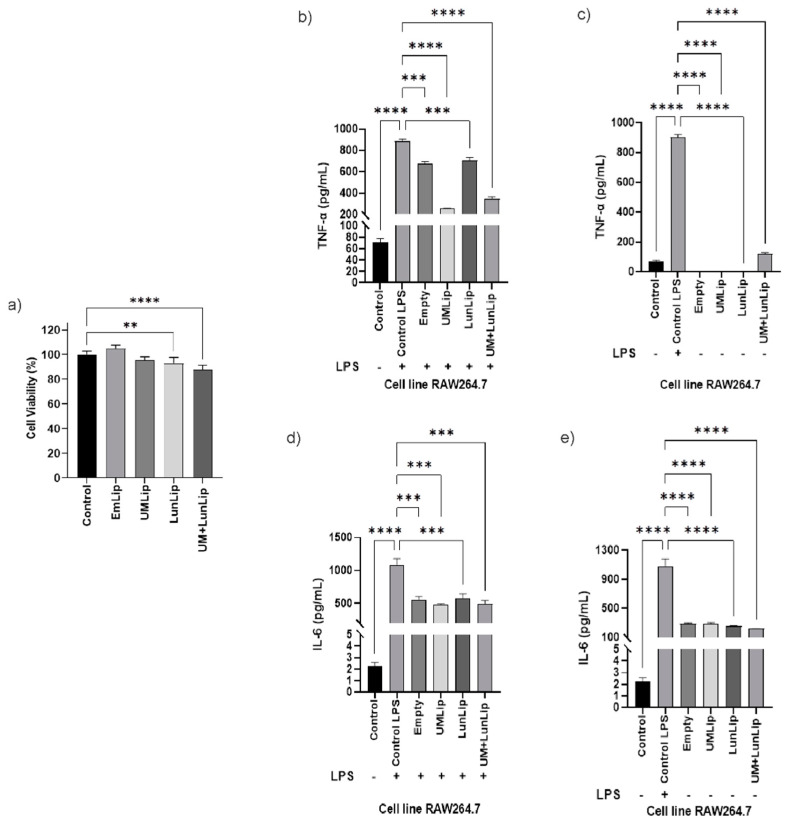
Liposomes prepared with dioleoyl phosphatidylcholine and dioleoyl phosphatidylglycerol, and loaded with soybean lunasin and amaranth unsaponifiable matter (UM + LipLun) decreased TNF-α and IL-6 production after LPS stimulation of RAW 264.7 macrophages. (**a**) Cell viability of RAW 264.7 macrophages after treatment with empty liposomes (EmLip), unsaponifiable matter-loaded liposomes (UMLip), lunasin-loaded liposomes (LunLip), and UM + LunLip. (**b**) TNF-α expression after treatment with liposomes to LPS-stimulated RAW 264.7 macrophages. (**c**) TNF-α expression after treatment with liposomes to non-LPS-stimulated RAW 264.7 macrophages. (**d**) IL-6 expression after treatment with liposomes to LPS-stimulated RAW 264.7 macrophages. (**e**) IL-6 expression after treatment with liposomes to non-LPS-stimulated RAW 264.7 macrophages. All the treatments consisted of 2 mg lunasin/mL or its equivalent in UMLip and EmLip.** *p* < 0.05; *** *p* < 0.001; **** *p* < 0.0001.

We hypothesized that UM + LunLip could reduce IL-6 and TNF-α production after the LPS-stimulation of macrophages. Cell viability was measured in peritoneal (Figure 3a) and RAW 264.7 (Figure 4a) macrophages to ensure that the cytokine production difference was due to the treatments and not because of a cytotoxic effect. When all the treatments were compared to the control, there was no difference in the cell viability of the peritoneal macrophages. In contrast, there was a difference (*p* < 0.01) comparing LunLip and UM + LunLip with the control in RAW 264.7 macrophages. Even though there was a statistical difference, the cell viability was 88% when the RAW 264.7 cells were treated with UM + LunLip and 93% when treated with LunLip.

In both cell lines, UMLip was the most effective treatment for TNF-α. Compared to the LPS-stimulated control, UMLip reduced TNF-α production by 90% in the peritoneal macrophages (Figure 3b) and 71% in the RAW 264.7 cells (Figure 4b). Inflammatory and autoimmune diseases, including rheumatoid arthritis, osteoarthritis, asthma, inflammatory bowel disease, and others are caused by TNF-α production. TNF-α overproduction is thought to promote chronic inflammation [7].

IL-6 behaves similarly to TNF-α; UMLip and UM + LunLip were the most effective treatment in both cell lines. In the peritoneal macrophages, UMLip and UM + LunLip reduced IL-6 production by 43% and 50%, respectively, in comparison to the LPS-stimulated control (Figure 3d). While in RAW 264.7, production decreased by 55% and 54%, respectively (Figure 4d).

It is important to mention that the liposome bilayer was composed of two molecules of oleic acid esterified to the glycerol. As shown (Figure 3 and Figure 4b–d), EmLip decreased the expression of TNF-α and IL-6 in both cell lines. Similarly to our results, in an in vivo acute kidney injury model, animals were treated using oleic acid, an unsaturated fatty acid, at either 10 or 30 mg/kg decreasing the production of IL-6 and TNF-α [32].

The application of TNF-α to cardiomyocytes produced a decrease in cell viability by activating pro-apoptotic proteins; however, when oleic acid was applied, pro-apoptotic protein expression was mitigated and oxidative stress was decreased [33]. In LPS-stimulated THP-1 monocytes, it was shown that the treatments with oleic acid decreased TNF-α and IL-6 production by inhibiting NF-κβ [34].

Our results suggest that the amaranth unsaponifiable matter loaded into liposomes is effective in reducing the inflammation markers TNF-α and IL-6, rather than lunasin; since LunLip in both cell lines behaved similarly to the empty liposomes. Similarly, Kao et al. [7] loaded squalene in liposomes at 1 mg/mL and treated LPS-stimulated RAW 264.7 with different squalene concentrations. They reported the inhibition of the production of both cytokines after the treatments. The application of squalene from olive oil unsaponifiable fraction to human monocytes diminished the production of IL-6, TNF-α, and other markers in a concentration-dependent manner [13].

Although some authors had reported a decrease in cytokines production when treating RAW 264.7 cells with up to 1.10 mg/mL lunasin per 24 h [15,30,35], the current study found that when LunLip was applied, IL-6 and TNF-α production decreased at the same level as EmLip; this was the case even though the treatment time was only 18.5 h.

Inflammation can contribute to angiogenesis, metastasis, antitumor immune response, and reaction to chemotherapeutics agents [36]. Moreover, inflammation has been related to poor prognosis in cancer patients; and cytokines (IL-6 and TNF-α) play a role in melanoma progression. IL-6 has been related to angiogenesis in melanoma tumors [37]. Additionally, we have previously demonstrated that UM + LunLip is effective against melanoma skin cancer [11]. The results from this research showed that there is a decrease in the production of ROS and the pro-inflammatory markers IL-6 and TNF-α; therefore, this behavior can influence the anticancer properties of the liposomes when applied to an in vivo model.

The scientific value of this manuscript was, to our knowledge, to demonstrate for the very first time that the anti-inflammatory potential of amaranth unsaponifiable matter loaded in liposomes decreased ROS, IL-6, and TNF-α in LPS-stimulated macrophages; suggesting the anti-inflammatory potential of the nanoparticles, as well as the potential anticancer effect previously reported using these liposomes [11,38].

## 4. Conclusions

The higher ROS production decrement was due to the higher dose of UM + LunLip treatments in fibroblasts CHON-002. Unsaponifiable matter loaded into liposomes (UMLip) was effective in decreasing the markers of inflammation TNF-α and IL-6 produced by LPS-stimulated RAW 264.7 and peritoneal macrophages.

In addition to the encapsulated compounds, the phospholipids in the liposome’s bilayer helped as well to decrease the production of the pro-inflammatory markers IL-6 and TNF-α; this was due to the presence of oleic acid. It would be interesting to test empty liposomes with an additional phospholipid.

## Data Availability

Not applicable.
